# A Case of Paraneoplastic Myoclonus Attributed to Non-Small Cell Lung Cancer

**DOI:** 10.5334/tohm.42

**Published:** 2020-06-16

**Authors:** Jennifer L. Nichols, Brett J. Lee, Kathryn A. Chung

**Affiliations:** 1 Oregon Health and Science University (OHSU) and the VA Portland Health Care System, US

**Keywords:** paraneoplastic, myoclonus, non-small cell lung cancer, adenocarcinoma, papillary adenocarcinoma

## Abstract

**Background:**

It is well known that myoclonus can be a paraneoplastic manifestation of underlying malignancy.

**Case Report:**

A 78-year-old male diagnosed with papillary variant non-small cell lung cancer (NSCLC) presented with tremulousness that rapidly evolved into severe, diffuse myoclonus with prominent palatal involvement requiring intubation. The generalized myoclonus resolved with on levetiracetam, chemotherapy and immune modulation. While low titer positive P/Q type calcium channel autoantibodies were detected, it’s etiologic relevance is unclear.

**Discussion:**

This case highlights a rare neurologic paraneoplastic presentation of papillary NSCLC. It also illustrates the importance of monitoring airway safety when myoclonus is generalized.

**Highlights:**

A new, rare paraneoplastic presentation of papillary variant non-small cell lung adenocarcinoma is described. The patient presented with severe diffuse myoclonus with prominent palatal involvement without encephalitis that responded to a combination of chemotherapy, immune modulation, and levetiracetam. No clear causal antibody was found.

## Introduction

Myoclonus is often caused by metabolic disruptions or pharmaceutical side effects, but has also been described as part of paraneoplastic presentations with only a few known antigens. Opsoclonus-myoclonus syndrome is associated with the ANNA-2 antigen (anti-Ri) in small cell lung cancer, neuroblastoma and ovarian and breast cancer. Myoclonus, tremors, ataxia and parasomnia has been associated with the DPPX antigen in leukemia and lymphoma.

Non-small cell lung cancer (NSCLC) accounts for 80% of primary lung malignancies. Adenocarcinoma, a subtype, has been linked to neurologic presentations including choreiform movement disorders [1]. One case of paraneoplastic polymyoclonus, cerebellar ataxia, and laryngospasm due to widespread metastatic adenocarcinoma with presumed lung origin is also described [2]. However, the adenocarcinoma papillary variant has only been associated with a paraneoplastic hematologic disease known as Evans syndrome [3] and never with neurologic manifestations.

## Case Description

We describe a patient with papillary non-small cell lung adenocarcinoma who developed isolated acute onset rapidly progressive myoclonus with prominent palatal involvement as a paraneoplastic manifestation.

A 78-year-old male with past medical history of coronary artery disease, hypertension and hyperlipidemia was diagnosed with papillary variant stage IIIB NSCLC after developing multiple pulmonary embolisms and was based on PET imaging and cervical lymph node biopsy.

Five months after the initial pulmonary embolism and one week prior to first planned chemotherapy treatment, patient was admitted with nausea and tremulousness. On the first examination day, the patient had irregular, high frequency, low amplitude diffuse action and postural tremor of the arms. It is possible that surface EMG may have reclassified this movement as polymyoclonus [4], however by the following 24 hours, it evolved to high amplitude near-constant spontaneous and stimulus-induced rhythmic myoclonus involving all extremities with proximal and right sided predominance. His pectoral muscles and palate were also prominently involved. Mental status remained intact.

MRI with and without contrast of brain and entire spinal cord was unremarkable. Metabolic and infectious lab workup was unrevealing. Lumbar puncture was only significant for a mildly elevated protein (61mg/dL; high normal 45mg/dL). CSF oligoclonal bands, IgG index, cytology, lymphocyte/leukemia markers and paraneoplastic panel were unremarkable. Notably, a serum paraneoplastic panel revealed positive P/Q type Calcium channel blocker antibody (0.09nmol/L; reference normal value <0.02nmol/L).

After ruling out infection, patient received 1gm methylprednisolone daily for three days. After no improvement, he received his first dose of carboplatin and pemetrexed with dexamethasone. Myoclonus including palatal myoclonus continued to worsen such that he could not hold objects and could not protect his airway, resulting in an aspiration event requiring intubation. The patient was given another three-day course of 1gm daily IV methylprednisolone with concurrent propofol (30–40 mcg/kg/min), baclofen (max 10 mg tid) and clobazam (5 mg bid) without improvement. EEG showed diffuse background slowing with brief runs of generalized periodic discharges consistent with cortical excitability. He was titrated off clobazam and baclofen and placed on levetiracetam 1.5g BID. This resulted in improvement in both the EEG and patient’s myoclonus. At discharge only a mild action tremor in his upper extremities remained.

## Discussion

This patient presented with rapid onset of generalized myoclonus in which the etiology was likely paraneoplastic in nature. Other causal considerations included toxic-metabolic syndromes, which were ruled out with detailed history and laboratory investigations, as were unlikely causes such as inherited or epileptic conditions given the age of onset. Other neurodegenerative conditions were deemed less likely given the known presence of an active tumor.

Treatment of Paraneoplastic Neurologic Syndromes (PNS) is largely based on expert opinion; there are no clinical trials to compare efficacy. The mainstay is treating the underlying primary malignancy, which is supported by multiple case series that identified this as the main factor leading to stabilization or improvement of PNS [5]. Given chemotherapy and radiation often have delayed effects, additional immune modulating treatments are often used concurrently to treat PNS. A large European PNS series found that the most frequently used treatments for PNS were corticosteroids (33.4%), IVIG (22.9%), plasma exchange (7.2%) and immunosuppression agents such as rituximab and mycophenolate mofetil (6.4%). The same series found that the most frequently used tumor treatments were chemotherapy (51.2%), surgery (30.0%) and radiation therapy (23.7%) [6]. In the presented case, a combination of chemotherapy, anticonvulsants and corticosteroids were used to successfully treat both the primary malignancy and associated PNS.

While this patient’s cancer was recognized before the PNS, it is more common for the PNS to precede an oncologic diagnosis. It is unlikely the patient’s positive P/Q type Calcium channel blocker antibody was directly related to his neurologic symptoms. This antibody has been historically associated with Lambert-Eaton syndrome with or without opsoclonus-myoclonus, cerebellar degeneration and less commonly encephalomyeloneuropathic syndromes [7,8]. It is more likely this finding was indicative of disordered immune regulation.

Regardless, this patient’s presentation of generalized severe myoclonus with prominent palatal involvement without encephalitis is to our knowledge, the first report of a paraneoplastic neurologic syndrome associated with papillary lung adenocarcinoma, culminating in a good response to oncologic and immune modulating therapies. An alternate explanation for the rarity may be that the patient had mixed pathology tumor undetected during the histologic exam, and that a region of small cell carcinoma (which is far more commonly associated with PNS) was responsible.

While this case points to the danger of severe generalized symptomatic myoclonus affecting the palate, which can lead to aspiration due to compromised airway protection, it also draws attention to myoclonus as the sole paraneoplastic presentation of NSCLC and highlights the need for continued exploration into efficacious treatments for PNS.
